# A Medical Sensation

**Published:** 1896-11

**Authors:** 


A MEDICAL SENSATION.—A Dr. Luys, of Paris, a pupil of the late great Charcot, and on whom Charcot's mantle has fallen, it is said, according to a correspondent in the *N. Y. Journal*, has made a remarkable discovery in hypnotism. He has two women-subjects who are very susceptible to the hypnotic influence, and one of them being in the lethargic state of hypnotism, reclining in a chair, attracted the doctor's attention as he passed her in the ward. Her head had fallen to one side, and he attempted to set it straight with his right hand. In his right hand he had some *pulv. ipecac* in a vial. Upon bringing his hand in contact with the subject's head, he was alarmed to see the features change to an expression of fright or horror; the pupils dilated, the mouth drawn, and the throat swollen to an enormous size. This condition changed upon removing his hand. Experiments led to the discovery (?) that medicines can be administered in this way,—during the hypnotic trance,—that is, "in the neck"; but the strange part of it is, that the characteristic effects of the medicine are not produced; except that of morphia; morphia producing sleep, as when otherwise administered. It is claimed by the *N. Y. Journal* that a New York doctor has verified Dr. Luys' discovery. But as we never heard of the doctor named by the *Journal*, and as the *Journal* is nothing if not sensational, we rather suspect that there is something suggestive in the name of the discoverer,—according to how it is pronounced;—L-u-y-s "lies."

